# Dual Role of Novel Ingenol Derivatives from *Euphorbia tirucalli* in HIV Replication: Inhibition of *De Novo* Infection and Activation of Viral LTR

**DOI:** 10.1371/journal.pone.0097257

**Published:** 2014-05-14

**Authors:** Celina M. Abreu, Sarah L. Price, Erin N. Shirk, Rodrigo D. Cunha, Luiz F. Pianowski, Janice E. Clements, Amilcar Tanuri, Lucio Gama

**Affiliations:** 1 Departamento de Genética, Universidade Federal do Rio de Janeiro, Rio de Janeiro, Brazil; 2 Department of Molecular and Comparative Pathobiology, Johns Hopkins University School of Medicine, Baltimore, Maryland, United States of America; 3 Kyolab, Campinas, Brazil; University of California, San Francisco, United States of America

## Abstract

HIV infection is not cleared by antiretroviral drugs due to the presence of latently infected cells that are not eliminated with current therapies and persist in the blood and organs of infected patients. New compounds to activate these latent reservoirs have been evaluated so that, along with HAART, they can be used to activate latent virus and eliminate the latently infected cells resulting in eradication of viral infection. Here we describe three novel diterpenes isolated from the sap of *Euphorbia tirucalli,* a tropical shrub. These molecules, identified as ingenols, were modified at carbon 3 and termed ingenol synthetic derivatives (ISD). They activated the HIV-LTR in reporter cell lines and human PBMCs with latent virus in concentrations as low as 10 nM. ISDs were also able to inhibit the replication of HIV-1 subtype B and C in MT-4 cells and human PBMCs at concentrations of EC_50_ 0.02 and 0.09 µM respectively, which are comparable to the EC_50_ of some antiretroviral currently used in AIDS treatment. Control of viral replication may be caused by downregulation of surface CD4, CCR5 and CXCR4 observed after ISD treatment *in vitro*. These compounds appear to be less cytotoxic than other diterpenes such as PMA and prostratin, with effective dose versus toxic dose TI>400. Although the mechanisms of action of the three ISDs are primarily attributed to the PKC pathway, downregulation of surface receptors and stimulation of the viral LTR might be differentially modulated by different PKC isoforms.

## Introduction

The most redoubtable obstacle to the eradication of HIV is the persistence of latent virus in infected patients under antiretroviral therapy (ART). Highly active antiretroviral therapy (HAART) targets only actively replicating virus and it has little influence on latent viral reservoirs. Despite the effectiveness of HAART on HIV replication, patients cannot be cured due to establishment of viral latency in long-life immune cells such as resting CD4+ T cells and macrophages [1]–[4].

HIV eradication in virally suppressed patients would require elimination or inactivation of these viral reservoirs; it is estimated that decades of HAART would be required for depletion of the reservoir source [5]. Therefore, agents that can safely facilitate purging of the latent virus could reduce the time for depletion when used in combination with HAART. The focus of most therapeutic strategies has been on eliminating this HIV reservoir to diminish the number of latently infected cells and to achieve a functional or even a sterilizing cure. A potentially useful strategy, sometimes termed “shock and kill” [6], [7], aims to attack the latent reservoir by treating patients with HIV-activating agents to stimulate viral replication in latently infected cells while blocking *de novo* infection with antiretroviral therapy. An ideal drug to induce HIV-LTR should be potent, orally available, nontoxic, active in a wide variety of latently infected cell types, and capable of penetrating tissues, including the central nervous system [8].

HIV transcription requires a protein complex formed by components of the positive transcription elongation factor b (P-TEFb) and the virally encoded Tat, which then associates with the Tat-activating region in the nascent transcript of HIV *gag* [9]. Events that contribute to HIV transcriptional latency include repressive chromatin structure, transcriptional interference, the inability of Tat to recruit P-TEFb complex, absence of interaction of nuclear factors with P-TEFb complex, and poor processivity of RNA polymerase II (RNAP II) [10]–[12]. Therefore, one approach to remove latently integrated HIV from CD4+ T cells is targeting the provirus epigenetic environment. The first cellular factors shown to be able to activate HIV-LTR were cytokines, mainly IL-2, IL-7 and TNF-α [13]–[15]. These molecules, however, must be carefully used since their administration can lead to cytokine storm [16].

Several synthetic compounds have been suggested for the “shock and kill” strategy, including histone deacetylase inhibitors (HDACIs), histone methyltransferases (HMTs), DNA methyltransferase inhibitors (DNMTIs) and protein kinase C (PKC) activators [17], [18].

Various natural and synthetic compounds - mainly diterpenes - are able to activate PKC isoforms by binding to its regulatory domain, mimicking the physiologic ligand diacylglycerol (DAG) [19], [20]. These compounds rapidly traffic through the cell membrane and are able to upregulate the HIV-LTR mainly via NF-κB and AP-1 signaling pathways [21]. Member of the phorbol ester family, such as 12-deoxyphorbol 13-phenylacetate (DPP), phorbol 13-myristate acetate (PMA), and 12-deoxyphorbol 13-acetate (prostratin) have been reported to efficiently activate the HIV and SIV LTR [8], [22]–[26]. Other non-phorbol compounds, such as the diterpene gnidimacrin and the macrocyclic lactone bryostatin-1, also activate PKC and are able to remove HIV from latency [27], [28]. Because PKC isoforms are involved in various cellular events, both phorbol and non-phorbol molecules have been reported to modulate pathways that indirectly interfere with viral replication. For instance, prostratin and gnidimacrin downregulate CD4 and CXCR4 expression in both cell lines and primary cells, preventing viral spread in these cultures [24], [25], [29], [30]. Similarly, bryostatin-1 decreases levels of surface CD4 and CXCR4 in peripheral blood T-lymphocytes, blocking HIV infection [31], [32]. Non-carcinogenic PKC activators, such as prostratin and bryostratin-1, were subsequently enrolled as potentially safe anti-HIV drugs, in contrast to tumorigenic PKC activators like PMA. However, major obstacles for further development of these compounds include limited availability, toxicity and low potency [33]–[35].

Additional candidate diterpenes for the eradication of HIV latent reservoirs were isolated from plants of the Euphorbia family and are structurally similar to other phorbol esters, while demonstrating non-carcinogenic properties [36]. One of these compounds, a tigliane diterpene termed ingenol, has been isolated from several Euphorbia species, including *E. escula, E. fischeriana, E. ingens* and *E. peplus* [19], [36]–[39]. They all share a common core ring, although present modifications in hydroxyl groups [40]. As with other diterpenes, they activate different PKC isoforms, driving NF-κB nuclear translocation that leads to HIV-LTR activation [41]–[44]. These novel compounds have been suggest as treatment for other medical conditions. For instance, ingenol mebutate (also called ingenol-3-angelate, isolate from *E. peplus*) causes necrosis of tumor cells via PKC pathways, and has been used as a medicinal gel (Picato) for the treatment of actinic keratosis [38], [39], [45]–[47].

Here we describe three novel ingenol compounds synthetically modified from natural isoforms isolated from *Euphorbia tirucalli*. These new PKC agonists were comparable to other diterpenes regarding HIV-LTR activation, and presented low levels of cytotoxicity. In addition, they reduced *de novo* HIV infection in cell lines and CD4+ lymphocytes, possibly through the downregulation of the viral CD4 receptor and CXCR4/CCR5 coreceptors. One of these compounds, ING-B, has been evaluated *in vivo* and presented low toxic profile (no-observed—adverse-effect level of 4 mg/kg/day) in rats and dogs determined by oral dosing (personal communication from Aurigon Life Science – Tutzing, Germany). Rhesus macaques orally treated with ING-B at similar dose also presented negligible side effects (personal communication from Bioqual – Rockville, USA), suggesting that these ingenol derivatives represent viable candidates to be considered as adjuvant treatment for HIV eradication.

## Methods and Material

### Ethics statement

Ethical approval for the collection of blood from both healthy and HIV-infected patients was granted by the Human Subject Ethics Committee for the Hemorio Center and Hospital Universitário Gaffrée e Guinle (Rio de Janeiro – Brazil). All patients provided written informed consent. Healthy donors were interviewed before blood collection by the Hemorio Center technicians, which were also responsible for the selection of subjects to be used in our study. HIV-infected patients were selected according to laboratorial characteristics (undetectable viral load and CD4+ T cell counts higher than 500 cells/mm^3^). Subjects were interviewed by a physician and informed about our study by one of our lab members. Blood was only collected after the patient signed a consent form.

### Ingenol synthetic derivatives (ISD)

**Figure 1:**
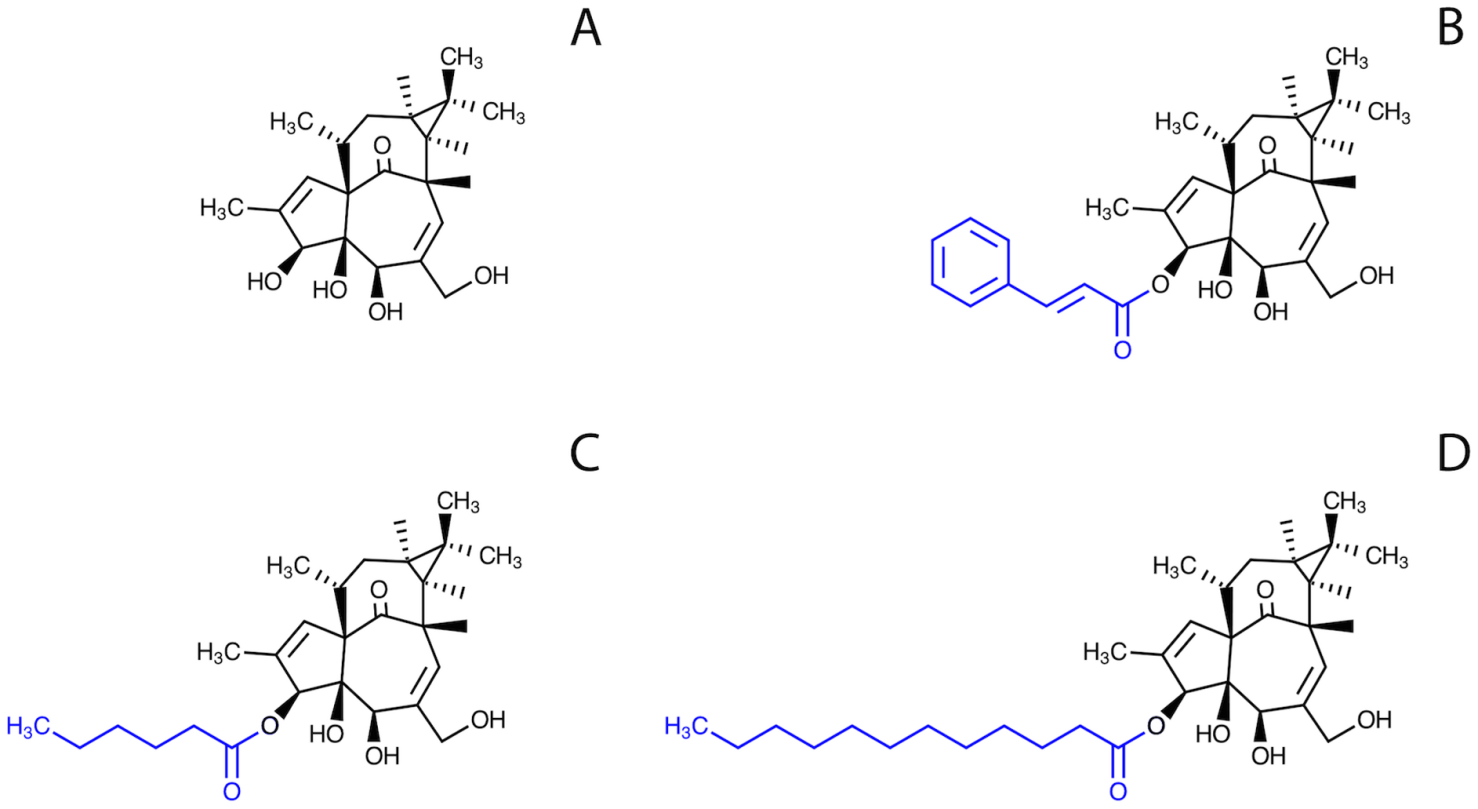
Chemical structures of ingenol synthetic derivatives indicating the modifications at carbon 3. A) Ingenol core; B) Ingenol-3-trans-cinnamate (ING-A); C) Ingenol-3-hexanoate (ING-B); D) Ingenol-3-dodecanoate (ING-C).

Novel ingenol synthetic derivatives originating from naturally occurring diterpenes extracted from the sap of *Euphorbia tirucalli* were modified by the addition of defined ester chains to the carbon 3 of the core ring, yielding three distinct molecules named ING-A (ingenol-3-trans-cinnamate), ING-B (ingenol-3-hexanoate) and ING-C (ingenol-3-dodecanoate) (Figure 1). Synthesis and modifications were done by Kyolab Laboratories (Campinas, Brazil). The three ingenol derivatives are experimental drugs generously provided by Amazônia Fitomedicamentos, Brazil (patent pending). All ISDs were diluted in DMSO (10 µM stock) and stored at −80°C.

### Human peripheral blood mononuclear cells (PBMCs) from healthy donors

Human samples from HIV-negative donors were either acquired as leukopacks (commercially) provided by the New York Blood Center (New York, USA) or as blood donations from the Hemorio Center (Rio de Janeiro, Brazil). PBMCs were isolated using a Ficoll-Paque Plus protocol (GE Healthcare, UK) and were cultured in RPMI 1640 medium (Life Technologies) supplemented with 10% fetal bovine serum (R10), 2 mM L-glutamine, 100 U/mL penicillin, and 100 mg/mL streptomycin at 37°C and 5% CO_2_ atmosphere.

### Cell Lines and Culture Conditions

The following cell lines were obtained through the NIH AIDS Reagent Program, Division of AIDS, NIAID: U1, J1.1 and ACH-2 from Dr. Thomas Folks; J-Lat clone (6.3, 8.4, 9.2, 10.6 and 15.4), from Dr Eric Verdin, MT-4 [48] from Dr. Douglas Richman, and were grown in R10 containing 50 U/mL of penicillin, 50 µg/mL of streptomycin, and 2 mM L-glutamine at 37°C and 5% CO_2_ atmosphere. 293LTV cells [49] were purchased from Cell Biolabs (USA) and were grown in DMEM (high glucose) (Life Technologies), 10% fetal bovine serum (FBS), 0.1 mM MEM Non-Essential Amino Acids, 2 mM L-glutamine, 50 U/mL penicillin, and 100 µg/mL streptomycin at 37°C and 5% CO_2_ atmosphere.

### Reagents and Plasmid

Prostratin, PMA, bryostatin-1 and PKC inhibitors (GÖ6983, GÖ6976, and RO-31-8220) were purchased from Sigma-Aldrich. Compounds were resuspended in DMSO and stored as recommended by the manufacturers. TNF-α was acquired from R&D Systems. HIV-derived vectors pNL4.3 and p-LTR-LUC, Efavirenz (EFV) and Zidovudine (ZDV) were obtained from the AIDS Reagent Program (NIAID). Another reporter vector, pNL4.3-Luc, was generously donated by Dr. Renato S. Aguiar (University of Rio de Janeiro). The construct was derived from pNL4.3-Luc E- R- (AIDS Reagent Program) and had the *env* stop codon reverted to the original sequence, therefore generating infectious particles upon transfection. The HIV-derived vector pZM247Fv-1 was generously donated by Dr. George M. Shaw from University of Pennsylvania School of Medicine [50].

### Cytotoxicity evaluation

Cytotoxicity of ISDs was evaluated in J-Lat (clones 9.2 and 10.6) and PHA-activated human PBMCs. Cells were added to wells in a 96-well plate in the presence of various concentrations of ISDs, PMA or prostratin in sextuplicates for 24 and 72 h. Cell viability was determined using the CellTiter-blue fluorescence Cell Viability Assay (Promega) following the manufacturer's instruction.

### Measurement of green fluorescent protein (GFP) expression in J-Lat clones

J-Lat cells are Jurkat cells stably transfected by an HIV genome whose *nef* sequence was replaced by the GFP gene. Also, a frameshift mutation was introduced to the *env* gene to prevent virus spread [51]. Because HIV LTR controls GFP expression, different J-Lat clones (6.3, 8.4, 9.2, 10.6 and 15.4) exhibit distinct activation profiles depending on the integration sites. To test the ISDs, a million cells of each clone were treated with different concentrations of each compound (ING-A, ING-B, ING-C, prostratin and PMA) at 37°C. TNF-α (10 ng/mL) was used as a positive control. After 48 h, cells were collected, washed with saline solution (PBS) and GFP expression was evaluated in a FACScalibur cytometer (BD Biosciences). Data were analyzed with Cell Quest Pro software (BD Biosciences).

### Measurement of gag p24 in U1, ACH-2 and J1.1 cell lines

ACH-2 and J1.1 are clonal cell lines derived from CEM [52] and Jurkat cells [53], respectively, after infection with HIV-1. U1 cells were derived from the human promonocyte cell line U937 and chronically infected with HIV-1 [54]. Each cell line (1×10^6^ cells) was incubated in the presence of 1 µM of diterpenes (ING-A, ING-B, ING-C, prostratin and PMA) or 10 ng/mL TNF-α for 48 h at 37°C. After incubation, cells were centrifuged and supernatants were collected for the quantitation of gag p24 antigen by ELISA, following the protocol described by the manufacturer (Perkin Elmer).

### Measurement of luciferase expression in primary human PBMCs

Human PBMCs were isolated from three healthy donors. Cells were resuspended in serum-free RPMI at 2×10^7^ cells/mL, and split in 0.4 mL aliquots mixed with 15 µg of the luciferase reporter plasmid pLTR-Luc. After electroporation (2.150 V, 20 ms pulse width), cells were resuspended in 6 mL of R10. After 12-hour incubation, cells were stimulated with each compound (ING-A, ING-B, ING-C, prostratin and PMA) for 24 h at 37°C. For the luciferase assays, cells were washed twice in PBS and lysed in 25 mM Tris-phosphate, pH 7.8, 8 mM MgCl_2_, 1 mM DTT, 1% Triton X-100, and 7% glycerol during 1 h at −80°C. The supernatants from cell lysates were used to evaluate the luciferase activity by a Glomax luminometer (Promega).

### Preparations of HIV-1 subtype B and C virus stock

pNL4.3 and pNL4.3-Luc (subtype B) and pZM247Fv-1 (subtype C) were transfected into 293LTV cells using lipofectamine (Life Technologies). Approximately 10^6^ 293LTV cells were seeded in 25 cm^2^ flasks 24 h before transfection. Medium was then removed and cells were incubated with 1.5 mL of transfection mixture containing 4 µg of each the respective plasmids and 10 µl of lipofectamine in Opti-MEM media (Life Technologies). Cells were incubated at 37°C for 5–6 h and then 8 mL of 10% FBS DMEM media was added to each flask. Virus-containing supernatants were collected 48 h later. Stocks were then centrifuged (2000 rpm for 10 min) and filtered through a 0.22 µm membrane (Millipore) to eliminate cell debris. Viral stock aliquots of 1 mL were stored at −80°C.

### Viruses Titration

Viral stocks were titrated in MT4 cells. Because HIV-1 (NL 4.3 and ZM247Fv-1) infection leads to cell death, infectivity was based of cell viability using CellTiter-blue fluorescence Cell Viability Assay (Promega) measured in a Spectramax M2 (Molecular Devices) at 560_ex_/590_em_ nm. Viral stocks generated from the pNL4.3-Luc construct were titrated by measuring luciferase activity.

### HIV infection in MT-4 cell line and human PBMCs

Human PMBCs were incubated with 100 U recombinant IL-2 (Sigma-Aldrich) and 5 µg/mL phytohemagglutinin-P (PHA-P) (Difco) for 5 days at 37°C. MT-4 cells (1×10^6^) or treated PBMCs (1.2×10^8^) were infected with HIV-1 clone NL-4.3 or ZM247Fv-1 (MOI of 0.001) by spinoculation (1200 g at room temperature for 2 h). Uninfected cells were used as negative control and were also subjected to the spinoculation process. After spinoculation, supernatants were discarded and cells were resuspended in 10 mL of R10. Cells from each condition were then added to wells (96-well plate) containing serial dilutions of each ISD. For MT-4 cells, each dilution was done in sextuplicates and, for PBMCs, in triplicates. Cells were also incubated with different concentrations of ZDV as positive control. After 5 days in culture, Cell Titer Blue was added to each well and cells were incubated for additional 24 h. The median effective concentration for each drug is defined as EC_50_. In parallel with the infectivity assay, the same cell types were treated with different concentrations of drugs to determine their cytotoxicity, following the protocol described above. Drug concentration that results in a 50% decrease in viable cells is defined as the CC_50_ for each compound. EC_50_ was calculated according to Spearman-Karber and therapeutic index was defined by the ratio between CC_50_ and EC_50_ [55].

### Luciferase expression in CD4+ T cells infected with NL 4.3-Luc

CD4+ T cells were isolated by negative selection from human PBMCs (2.5×10^8^ cells) using magnetic beads (Miltenyi) and incubated with 5 µg/mL PHA-P and 100 U IL-2 for 5 days. Cells were then seeded in 6-well plates (10^6^ cells per well) and treated with different concentrations of ING-B. After 24 h, cells were infected with HIV-1 NL-Luc (MOI of 0.1) by spinoculation. Infected cells were kept at 37°C for 24 h and then collected for luciferase activity evaluation as described above.

### Cytometry analysis

For analysis of cell surface marker expression, MT-4 cells and bead-selected CD4+ T cells (described above) were incubated with 1 µM of each compound (ING-A, ING-B, ING-C, prostratin, PMA, and bryostatin-1) at 37°C for 48 h. After incubation, cells were washed with PBS and incubated for 20 min at room temperature with antibodies against CD3-Brilliant Violet 510 (clone OKT3 – Biolegend), CD4-Qdot 655 (clone 53.5 - Life Technologies), CXCR4-PE (clone 1265 - BD Biosciences), CCR5-PerCP-Cy5.5 (clone 3A9 - BD Biosciences), CD69-Pacific Blue (clone FN50 – BD Biosciences), CD38-FITC (clone AT-1 – Stem Cell Technologies), CD25-APC (clone 2A3 – BD Biosciences), HLA-DR-Qdot 605 (clone TÜ36 – Life Technologies) and a marker for annexin V-FITC (Life Technologies). Cells (both MT-4 cells and CD4+ T cells) were initially incubated for 2 h with different PKC inhibitors (GÖ6983, GÖ6976 or RO-31-8220). Then, ING-B, prostratin or PMA at 1 µM or TNF-α at 10 ng/mL were added to each condition and incubation was carried out for 24 h at 37°C. Similar protocol was used for the analysis of PKC inhibitors in J-Lat clones 9.2 and 10.6. GFP expression, surface marker expression and activation marker were evaluated on a LSR Fortessa cytometer (BD Biosciences). Data analysis was performed using FlowJo software (Tree Star).

### Cellular proliferation assay

Human PBMCs were stained with CFSE following manufacturer's instructions (Sigma-Aldrich). After staining, cells were washed twice in PBS and incubated with ING-B (1, 5 or 10 µM), 5 µg/mL PHA-P plus 10 U/mL IL-2 (as positive control), or DMSO 1% (as negative control). Cell proliferation was assessed for 8 days after stimulation, and relative proliferation index was calculated for all conditions.

### Cell cycle analysis

Human PBMCs and MT-4 cells were incubated at 37°C with different concentrations of ING-B for 48 h. After incubation, cells were collected, washed twice with PBS, and stained with 200 µl of propidium iodide (50 µg/mL, diluted in Hanks buffer with 1 mg/mL RNAse and 0.2% Triton X-100) for 15 min on ice before cytometry analysis.

### Virus reactivation in CD4+ T cells from HIV+ HAART-treated patients

Whole blood from five HIV+ patients at the Hospital Universitário Gaffrée e Guinle (Rio de Janeiro, Brazil) was collected for reactivation experiments. All individuals enrolled in the study voluntarily and provided written informed consent. Patients presented undetectable viral load (<50 copies/mL), more than 500 CD4+ T cells/mm^3^ and have been HAART-treated for at least 10 months (Table 1). PBMCs where isolated following a Ficoll-Paque Plus protocol (GE Healthcare, UK). CD4+ T cells were selected by magnetic beads as described above, and 10^6^ cells were incubated in R10 containing 1 µM of the testing compounds (ING-B, prostratin or PMA). DMSO 1% was used as negative control. The antiretroviral EFV (10 µM) was added to all conditions to prevent viral spread. After 24 h, cells were collected for RNA isolation using the All Prep Qiagen kit (Qiagen), as instructed by the manufacturer.

**Table 1:** Baseline characteristics of study subjects.

Patient ID	Age (year)	Sex	CD4+ T cell count (cells/mm^3^)	Viral Load (copies/mL)	Time on HAART* (months)	HAART regimen**
P1	36	F	509	< 50	10	ZDV, 3TC, EFV
P2	42	M	701	< 50	22	TDV, 3TC, EFV
P3	23	F	634	< 50	15	ZDV, 3TC, EFV
P4	32	F	532	< 50	20	TDV, 3TC, EFV
P5	47	M	658	< 50	18	ZDV, 3TC, EFV

(*) Time on HAART with documented continuous suppression of plasma viremia (< 50 copies/mL); (**) Drug abbreviations: ZDV, zidovudine; 3TC, lamivudine; EFV, efavirenz; TDF, tenofovir disoproxil fumarate.

### Quantitative PCR for HIV *pol*

Total RNA from CD4+ T cells was analyzed by RT-qPCR using primers and probe (Life Technologies) specific for the HIV-*pol* region, as following: forward primer Pol-F (5'-AATGGCAGTATTCATCCACAA TTT T-3'), reverse primer Pol-R (5'-GTCTACTATTCTTTCCCCTGCACTGT-3') and a minor groove binder (MGB) probe Pol-Pb (5'-VIC-ATC CCC CCT TTT CTT T-3'). All reagents for the RT-qPCR reactions were kindly provided by Dr. Rodrigo Brindeiro (Universidade Federal do Rio de Janeiro, Brazil) and Dr. Patrícia Alvarez (Biomanguinhos/FioCruz Institute, Brazil) and consisted of a proprietary master-mix designed by the Biomanguinhos Institute for the screening of blood donors at the Hemorio Center (Brazil). Reactions were carried out as a one-step protocol with the following cycle parameters: One cycle of 30 min at 51°C, one cycle of 10 min at 95°C, and 40 cycles of 30 sec at 95°C and 1 min at 60°C, using the ABI 7500 thermocycler (Life Technologies). Results were extrapolated out of a 5-point standard curve (10^1^ to 10^8^ copies/reaction) and presented as HIV *pol* copy eq./10^6^ cells.

### Statistical analysis

All statistical analyses were performed using program GraphPism version 6.0. Titrations for TCID50 infectivity were calculated by the Spearman-Karber method. Dose response curves to ISDs with HIV-1 subtype B and C were done using non-linear regression and were fit to the Hill equation.

## Results

### ISDs upregulate HIV-1 LTR-dependent transcription in reporter cell lines

To evaluate whether ISDs were able to activate the HIV LTR, the three compounds were tested in five J-Lat cell lines (clones 6.3, 8.4, 9.2, 10.6 and 15.4). In all experiments, J-Lat clones were exposed to different concentrations of each ISD and GFP expression was measured 48 h later by cytometry. Cells were also exposed to two previously identified phorbol esters (PMA and prostratin) for comparison, and also to TNF-α (10 ng/mL) as a positive control.

**Figure 2:**
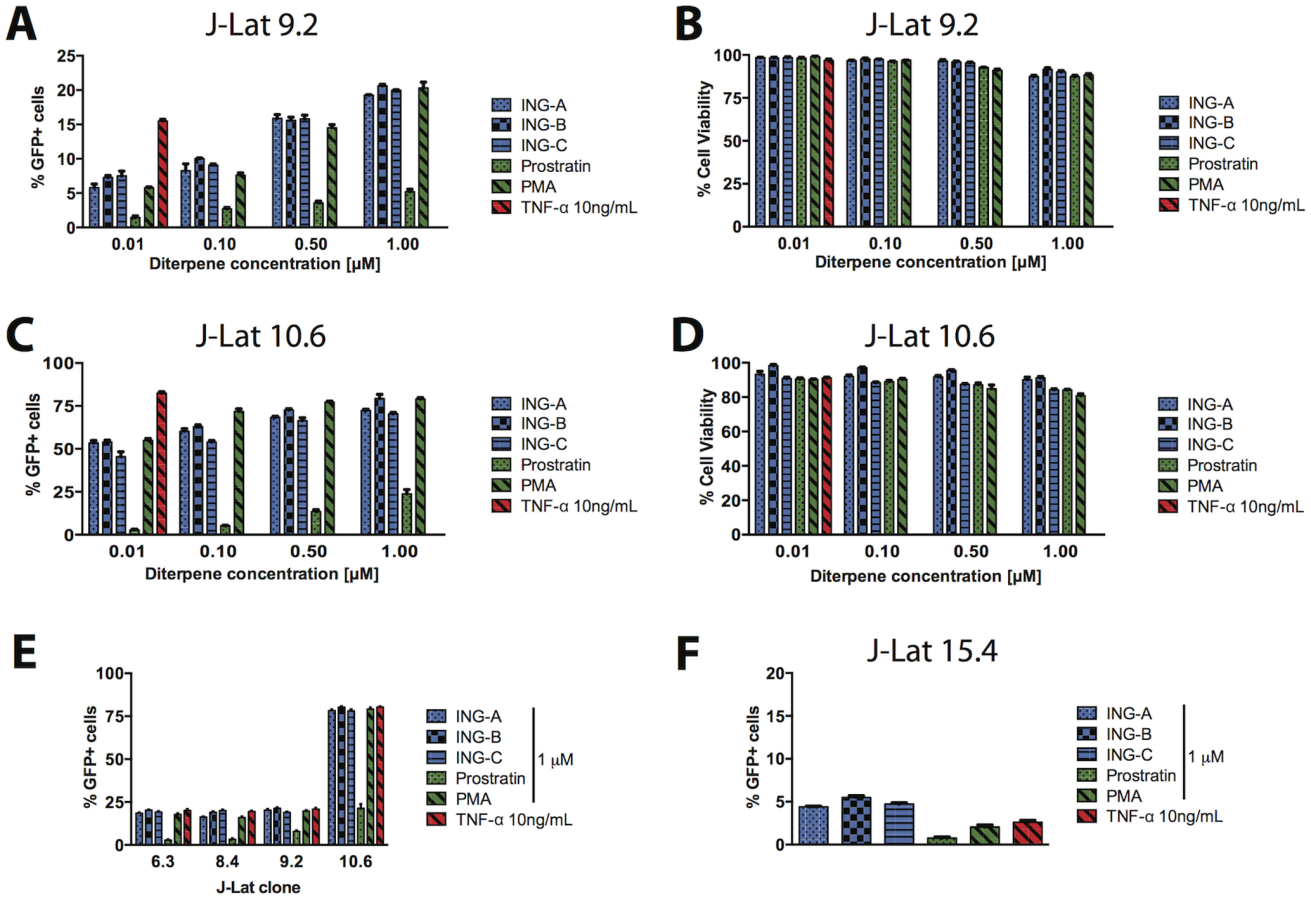
ISDs upregulate HIV-LTR-driven GFP expression in J-Lat clones. GFP expression (A) and viability (B) in J-Lat clone 9.2 were measured after cells were treated with ISDs, prostratin and PMA in different concentrations for 48 h. Similar experiment was done in J-Lat clone 10.6 (C and D). E) Comparison of GFP expression in four different J-Lat clones, all treated with 1 µM of each compound for 48 h. J-Lat clone 15.4 is depicted in a different graph (F). Graphs show mean and standard deviation of 3 separate tests. In all experiments, TNF-α 10 ng/mL was used as positive control.

All ISDs induced LTR-dependent GFP expression after 48 h post-treatment in all J-Lat clones, although the level of GFP induction varied according to the clone. Figure 2 (A–D) shows the results of clones 9.2 and 10.6 for both GFP induction and cell viability. ISDs, similar to PMA and in contrast, to prostratin, were able to activate the HIV LTR at nanomolar concentrations. Higher concentrations of ISDs led to levels of activation similar to TNF-α, a potent activator of HIV-1 latency in many cell line models of HIV latency [56], [57]. For both clones, levels of GFP expression followed a similar trend: PMA  =  ING-B > ING-A  =  ING-C > prostratin at all concentrations tested. ING-B was slightly more active than ING-A, ING-C and prostratin in clone 10.6, and achieved similar levels to PMA induction. Although cell viability was similar among the treatments, ING-B presented lower toxicity when compared to its counterparts (Figure 2 B and D). Similar LTR-driven GFP expression results were observed when the compounds were tested in clones 6.3 and 8.4. For the J-Lat clone 15.4, ISDs were able to activate the LTR to levels slightly higher than prostratin, PMA and TNF-α (Figure 2 E and F).

**Figure 3:**
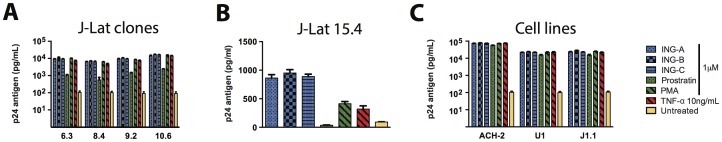
ISDs upregulate the production of HIV particles in diverse cell models. A) Four J-Lat clones were treated with 1 µM of different diterpenes and p24 levels in the supernatant were quantitated by ELISA. Similar experiment was done in J-Lat clone 15.4 (B) and other latent cell models (C). Graphs show mean and standard deviation of 3 separate tests. In all experiments, TNF-α 10 ng/mL was used as positive control.

To assess whether the GFP expression levels induced by ISDs in J-Lats led to viral production, all five clones were stimulated with 1 µM of each compound for 48 h and production of HIV-1 p24 was measured in the culture supernatant by ELISA. Levels of p24 were proportional to GFP expression in clones 6.3 and 9.2, whereas levels in clone 8.4 were lower when compared to GFP (Figure 3A). In contrast, after induction with ISDs, clone 15.4 showed levels of p24 higher than would be expected based on the GFP expression, being higher than PMA and TNF-α. Prostratin does not appear to work in this clone (Figure 3B). Taken together, these results show that ING-B is able to activate the HIV-LTR at equivalent levels to PMA and TNF-α and slightly higher levels than ING-A and ING-C. Conversely, prostratin appears to be the less effective compound among all tested.

Due to the complex mechanisms involved in HIV reactivation - from proviral insertional position to chromatin activation state - we also analyzed the effects of ISDs in other latency cell line models, including the T cell-derived ACH-2 and J1.1, and the promonocyte-derived U1. These cell lines contain one or two copies of integrated virus and constitutively display low levels of HIV-1 gene expression. ACH-2 cells displayed significantly higher levels of p24 production upon induction by all ISDs when compared to J1.1 and U1 cells (Figure 3C). p24 levels in ACH-2 after treatment with ING-B were closer to levels obtained with TNF-α (around 80,000 pg/mL), and higher than those with ING-A, ING-C and PMA. Cells stimulated with prostratin showed low levels of p24 production, corroborating previous reports [58] and suggesting that prostratin is less effective then ISDs in activating the HIV-LTR in these cell models. In J1.1 cells, ING-B at 1 µM led to as much p24 production as PMA and TNF-α, and twice as much as prostratin. All ISDs were also able to upregulate p24 production in the promonocytic cell line U1, similarly to PMA and TNF-α, and greater than prostratin. These results indicate that ISDs can activate HIV expression in both T-lymphocytes and myeloid cells, mimicking the different latently infected cells found in HIV-infected patients treated with HAART [59], [60].

### ISDs activate the HIV LTR in primary PBMCs

**Figure 4:**
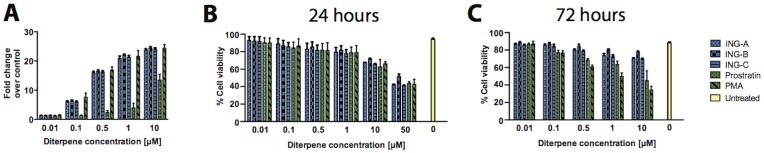
ISDs upregulate HIV-LTR-driven luciferase expression in human PBMCs. Blood-derived PBMCs from three healthy donors were transfected with pLTR-luc and then treated with different concentrations of ISDs, prostratin and PMA for 24 and 72 h. Graphs show levels of luciferase activity after 24 h (A) and cell viability after 24 (B) and 72 h (C). Results are represented as mean plus standard deviation of three independent experiments. DMSO 1% was used as vehicle control (untreated – yellow bar).

To examine whether ISDs could induce HIV-1 LTR expression in primary cells, PBMC isolated from three human donors were transfected with a plasmid containing the luciferase gene reporter under the control of the HIV-1 LTR promoter. PBMCs were then treated with different concentrations of ING-A, ING-B, ING-C, PMA and prostratin, and the expression of LTR-driven luciferase was evaluated 24 h post-treatment. Again, all ISDs were able to upregulate luciferase expression similarly to PMA, and in higher levels when compared to prostratin (Figure 4A).

In addition, all compounds presented similar cytotoxicity in PBMCs after a 24-hour incubation. However, ISDs were less toxic than the other diterpenes over time, as demonstrated at 72 h (Figure 4B and C). These results are similar to the latency cell lines model (Figures 2 and 3) and confirm that these novel phorbol-like compounds are as potent as PMA, but less toxic. All ISDs demonstrated similar cytotoxicity *in vitro* (Figure 4B), although ING-B appeared to be less cytotoxic after 72 h of treatment (Figure 4C). The results suggest that ING-B is a strong candidate for studies of activation of latency vivo, leading to a strong expression of genes under the control of the HIV-1 LTR in primary human cells and latently infected cell line models.

### ISDs inhibit HIV infection in MT-4 cells and human PBMCs

Some phorbol esters, such as prostratin and PMA, have been reported to inhibit HIV infection in lymphocytic cell lines. To assess whether ISDs also block *de novo* HIV infection, MT-4 cells and human PBMCs were infected with two different HIV-1 clones, NL4.3 (subtype B) and ZM247Fv-1 (subtype C). Immediately after infection, ISDs were added in different concentrations and cells were incubated for 6 days at 37°C. As positive control, cells were treated with ZDV with similar concentrations. The cytotoxicity of the three ISD was evaluated in parallel and the CC_50_ dose for all ISDs ranged from 25 to 40 µM for both cell types.

**Figure 5:**
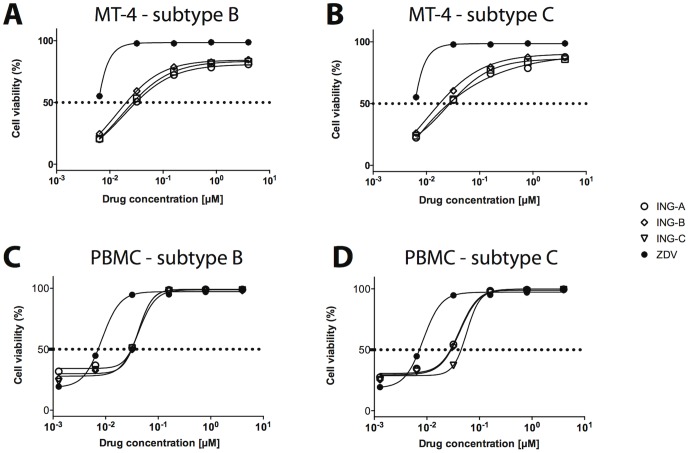
ISDs reduce HIV replication in a dose-dependent manner. Lymphocytic cell line MT-4 (A and B) and human PBMCs (C and D) were infected with HIV subtype B NL-4.3 (A and C) or HIV subtype C ZM247Fv-1 (B and D) and treated with different concentrations of ISDs. After six days, cell viability was evaluated using the Cell Titer blue kit. Curves were derived by non-linear regression (dose-response curve by Hill for 3 parameters), and dotted lines represent EC_50_. Experiments were done in triplicate for PBMCs and sextuplicate for MT-4. ZVD was used as positive control.

The drug control (ZDV) was very efficient in inhibiting HIV-1 subtype B and C replication in MT-4 and human PBMCs, with EC_50_ ranging from 0.004 to 0.008 µM. These values are equivalent to those previously described for ZDV in both cells [55] (http://chemdb.niaid.nih.gov). ISDs were able to block infection of HIV-1 subtype B and C in a dose-dependent manner, with values of EC_50_ close to 0.05 µM (Figure 5 and Table 2). Therapeutic indexes (TI) were calculated as the ratio between the concentration values for CC_50_ and EC_50_. The TIs for all ISDs for both MT-4 and PBMCs were within the range for low toxicity (TI>400) [61]. TI for ING-B was even higher (700 to 1200), suggesting that this compound is a potential candidate for *in vivo* studies.

**Table 2:** CC_50_ and EC_50_ of ISDs and ZVD in MT-4 cells and primary CD4+ T lymphocytes infected with different HIV subtypes (see Figure 5).

Compound	MT-4 cells						Human PBMC					
	Subtype B (X4)			Subtype C (R5)			Subtype B (X4)			Subtype C (R5)		
	CC_50_	EC_50_	TI	CC_50_	EC_50_	TI	CC_50_	EC_50_	TI	CC_50_	EC_50_	TI
**ING-A**	25	0.037	675.6	25	0.038	657.9	34	0.053	641.5	34	0.049	693.9
**ING-B**	28	0.022	1272	28	0.025	1120	40	0.056	714.3	40	0.047	851
**ING-C**	27	0.041	658.5	27	0.036	750	38	0.052	730.7	38	0.092	413
**ZDV**	NA	0.004^*^	NA	NA	0.005^*^	NA	NA	0.007	NA	NA	0.008	NA

CC_50_ and EC_50_ values are presented in µM. Therapeutic index (TI) was defined as the ratio CC_50_/EC_50_. NA: non applicable. (*) Value estimated by extrapolation.

### ISDs downregulate CD4, CXCR4 and CCR5 surface expression in MT-4 cells and CD4+ T cells

To investigate whether HIV replication suppression upon stimulation with ISDs was caused by downregulation of virus-specific surface receptors, MT-4 cells and human PBMCs were treated with 1 µM of each compound for 24 h and surface marker expression was analyzed by cytometry. Results are presented as fold change relative to cells treated with 1% DMSO. In this experiments we also evaluated bryostatin-1, a known macrocyclic PKC agonist that potently modulates CD4 and CXCR4 *in vitro*.

**Figure 6:**
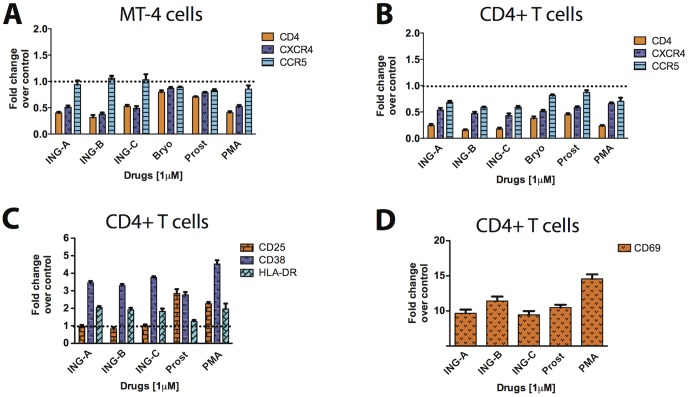
ISDs modulate surface expression of CD4, CXCR4, CCR5 and activation markers. MT-4 cells (A) and CD4+ T lymphocytes (B) were stimulated with ISDs (ING-A, ING-B, ING-C), bryostatin (bryo), prostratin (prost) and PMA for 48 h. Surface marker expression was evaluated by cytometry. CD4+ T cells were also evaluated for the activation markers CD25, CD38, HLA-DR (C) and CD69 (D). Results are representative of the mean and standard deviation of three independent experiments. DMSO 1% was used as vehicle control.

Levels of surface CD4 and CXCR4 expression in MT-4 cells were strongly downregulated by ISDs and PMA (down to 50% of that in the control condition), but not by prostratin and bryostatin-1. CCR5 expression was unaffected in all conditions, probably due to low expression on this marker in these cells (Figure 6A).

In CD4+ lymphocytes, all three surface markers were downregulated after treatment with ISDs. Specifically, levels of CD4 expression were severely diminished after ISD and PMA treatment when compared to prostratin and bryostatin-1. All compounds downregulated surface CXCR4 similarly and prostratin and bryostatin-1 were less efficient in downregulating surface expression of CCR5 (Figure 6B).

ISDs also activated CD4+ T lymphocytes, as demonstrated by the upregulation of specific activation surface markers. All diterpenes upregulated the early activation marker CD69 and the cyclic ADP ribose hydrolase (CD38) in a similar fashion, although PMA appeared do so more intensely when compared to the other compounds (Figure 6C–D). HLA-DR was upregulated by ISDs and PMA, but not by prostratin. Conversely, prostratin and PMA, but not ISDs, upregulated the surface expression of the alpha chain for the IL-2 receptor (CD25).

**Figure 7:**
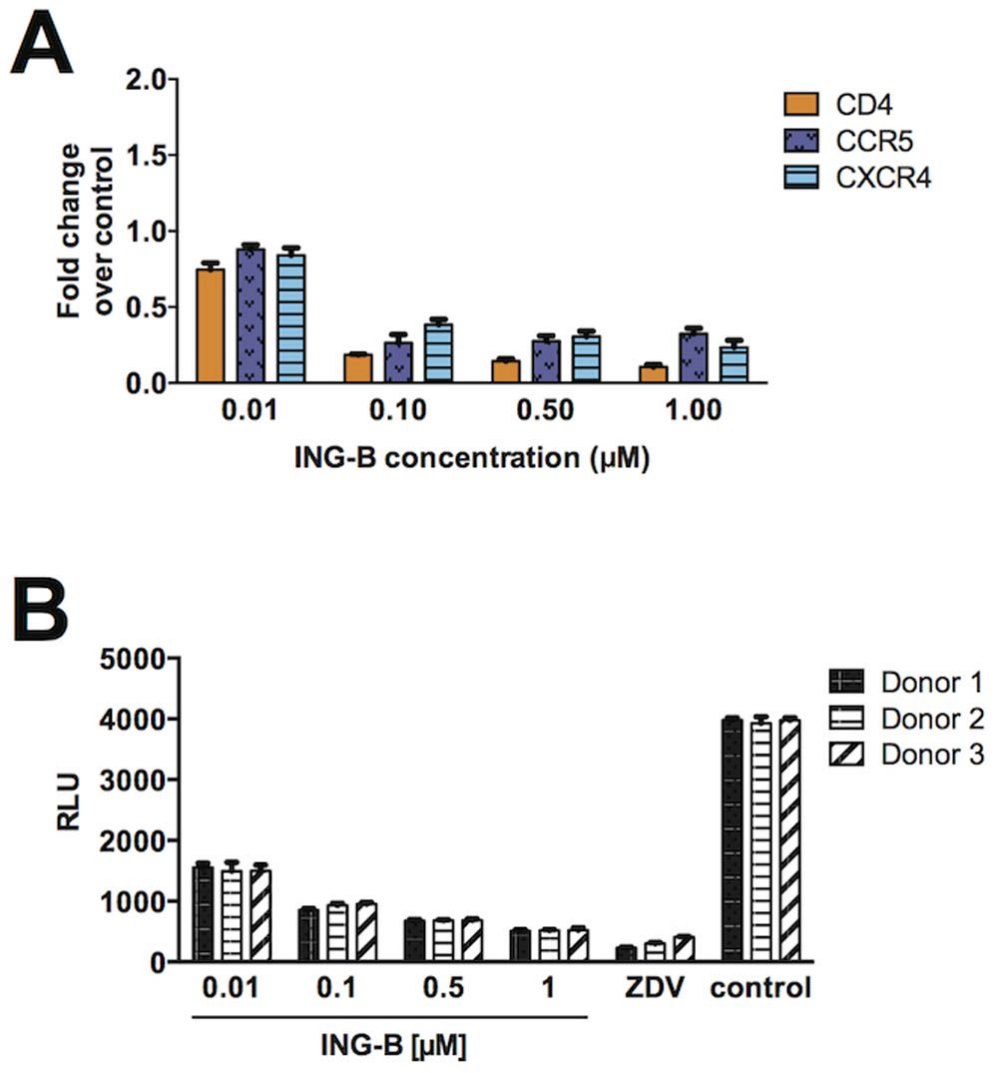
ING-B blocks HIV *de novo* replication through downregulation of surface receptors. CD4+ T cells from three healthy donors were stimulated with PHA/IL-2 for 5 days and then treated with different concentrations of ING-B for 24 h. A portion of the cells was used for cytometry evaluation (A), and the graph depicts the mean and standard deviation of three independent experiments. The remaining cells were infected with HIV NL4-3-Luc for 24 h and cell lysates were analyzed by luciferase activity (B). Results are shown as relative light units (RLU). ZVD 1 µM was used as positive control and DMSO 1% as vehicle control. Results are representative of mean and standard deviation of triplicates for each blood donor.

To evaluate the impact of CD4, CXCR4 and CCR5 decreased expression in HIV-1 *de novo* infection, we treated human PBMC isolated from three donors with different concentrations of ING-B for 24 h. After incubation, half of the cells were analyzed by cytometry, and the other half were infected by spinoculation with NL4.3-Luc (MOI of 0.1). Figure 7 shows the effect of ING-B on CD4, CCR5 and CXCR4, confirming that the downregulation of surface markers is dose dependent. Accordingly, levels of HIV-driven luciferase were dramatically reduced in cells pre-treated with ING-B, suggesting that viral entry was, at least partially, hindered by the presence of the compound.

### ING-B does not cause cell proliferation or early apoptosis

We further determined whether ING-B could increase cellular proliferation comparably to other diterpenes such as PMA. CSFE-stained human PBMC from healthy donors were treated with 1, 5 or 10 µM ING-B for 8 days and evaluated by cytometry (Figures S1A and B). There was no evidence that ING-B caused cell proliferation – even in the highest concentration of 10 µM, in contrast to the polyclonal mitogenic combination PHA plus IL-2, which gradually caused an increase in cell number to levels up to 20 fold higher than unstimulated. Similarly, ING-B treatment did not alter cell cycle phases in MT-4 cells. A slight change was observed in Sub G1 and G0, but it was not significant or dose-dependent (Figures S1C and D). In addition, apoptotic events in human PBMC were evaluated by cytometry using annexin-V. ING-B treatment led to levels of staining similar to those found in untreated cells (Figure S1E).

### ING-B activity is blocked by PKC inhibitors

**Figure 8:**
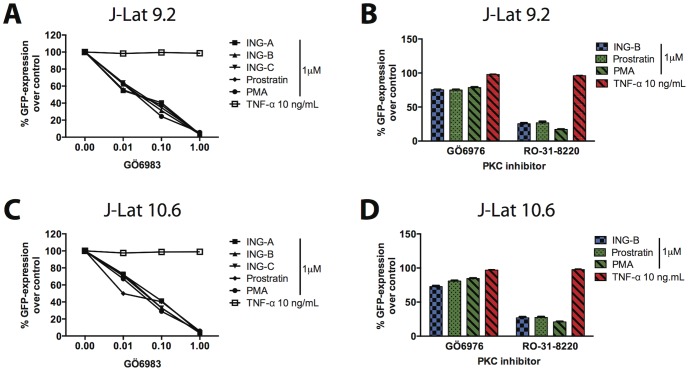
ISDs upregulated HIV-LTR-driven GFP expression through PKC pathways. J-Lat clones 9.2 (A and B) and 10.6 (C and D) were incubated with different PKC inhibitors prior to stimulation with ISDs and other compounds. Graphs A and C depict levels of relative GFP expression for J-Lat clone 9.2 (A) and 10.6 (C) after treatment with different concentrations of GÖ6983 and subsequent compounds. Graphs B and D show the relative GFP expression for J-Lat clone 9.2 (B) and 10.6 (D), after treatment with two PKC inhibitors (GÖ6976 and RO-31-8220) at 0.1 µM followed by treatment with ING-B, prostratin, PMA or TNF-α. Results are representative of mean and standard deviation of three independent experiments.

To investigate whether the effects of ING-B is PKC-dependent, J-Lat 9.2 and 10.6 clones were pre-treated with well-characterized PKC inhibitors, and then exposed to ING-B for 24 h. Levels of GFP expression were further analyzed by cytometry. Initially we tested GÖ6983, which has been shown to inhibit PKCζ in addition to conventional and novel PKCs isoforms such as PKCα, PKCβ, PKCγ, PKCθ and PKCδ. TNF-α (10 ng/mL) was used as control, since its effects are PKC-independent. GÖ6983 strongly blocked GFP expression in J-Lat clones 9.2 and 10.6 in a dose dependent way, implicating a PKC-dependent pathway for ING-B activity. Prostratin and PMA were also inhibited by GÖ6983, confirming that these diterpenes share the same pathway with ISDs (Figure 8A and C).

The PKC family of protein kinases includes classical, novel, and atypical subclasses. To verify which specific PKC subclass mediates the effects of ISDs on LTR activation, we treated J-Lat 10.6 cells with RO-31-8220 (which mainly impairs the activity of PKC-α, PKC-βI, PKC-βII, PKC-γ, and PKC-ε) and GÖ6976 (which inhibits the action of some conventional Ca^2+^-dependent PKC such as PKC-α and PKCβI, and does not inhibit isoforms from the novel and atypical PKCs). Pre-incubation with 0.1 µM RO-31-8220 reduced GFP expression to 75% in response to ISDs, prostratin and PMA, indicating that ING-B acts through convectional and/or novel subclasses of PKCs. In contrast, the addition of 0.1 µM GÖ-6976 had little effect on ISD induction of GFP expression (Figure 8B and D).

**Figure 9:**
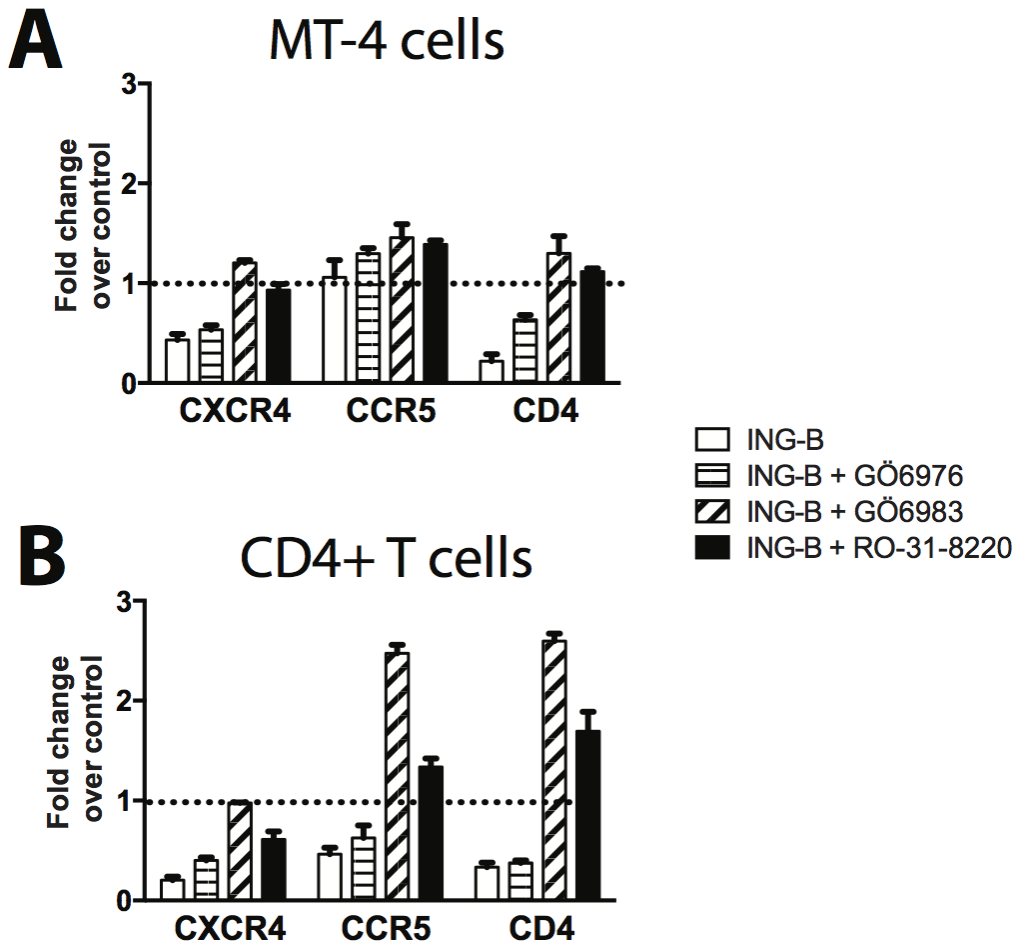
PKC inhibitors block the downregulation of surface receptors induced by ING-B. MT-4 cells (A) and human CD4+ T lymphocytes (B) were treated with 0.1 µM of each PKC inhibitor (GÖ6983, GÖ6976 and RO-31-8220) and then stimulated with ING-B 1 µM for 24 h. Cells were analyzed for surface marker expression by cytometry. Results are shown as fold change over vehicle control (DMSO 1%), and represent the mean and standard deviation of three independent experiments.

We next examined the potential contribution of distinct PKC isoforms to the downregulation of CD4, CCR5 and CXCR4 in response to ING-B stimulation. MT-4 cells and CD4+ lymphocytes were treated with 0.1 µM of each PKC inhibitors (GÖ6983, GÖ6976 and RO-31-8220) prior to 24-hour stimulation with 1 µM ING-B. When MT-4 cell were treated with Pan-PKC inhibitors (GÖ6983 and RO-31-8220), ING-B was not able to downregulate the expression of CD4 and CXCR4; however no effect on CCR5 expression was observed (Figure 9A). An interesting observation is that, in CD4+ T Lymphocytes, exposure to PKC inhibitors GÖ-6983 and RO-31-8220 in the presence of ING-B led to an overexpression of CD4 and CCR5 up to 2.5-fold when compared to untreated control, suggesting that the combination of these two chemicals may affect other cellular events related to the PKC pathway (Figure 9B). ING-B was unable to downregulate CXCR4 expression in the presence of GÖ-6983 and RO-31-8220 in MT-4 cells (Figure 9A). As it was observed for GFP expression, GÖ6976 had little effect on blocking receptor downregulation driven by ING-B. These experiments with PKC inhibitors revealed that PKC isoforms δ, βII, y, ζ, θ and ε may be the main ING-B mediators for HIV-LTR activation and CD4, CCR5/CXCR4 downregulation in cell lines and CD4+ lymphocytes.

### ING-B increases HIV transcription in cells isolated from virally suppressed HIV+ patients

**Figure 10:**
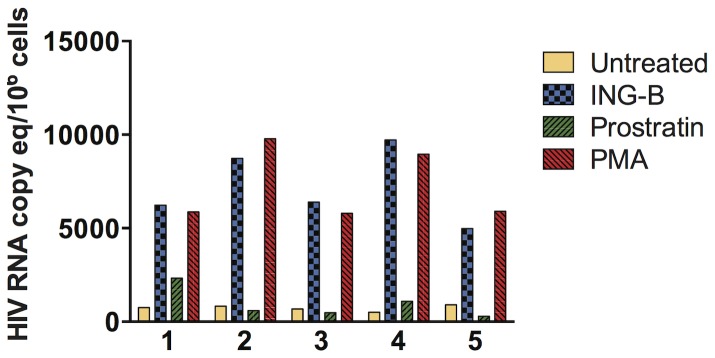
ING-B activates the HIV-LTR in cells isolated from virally suppressed HIV+ patients. PBMCs isolated from five HIV+ patients were treated with 1 µM of ING-B, prostratin or PMA for 24 h. Total RNA was isolated and HIV *pol* RNA was quantitate by RT-qPCR. All patients were HAART-treated and presented undetectable levels of plasma viral load.

*In vitro* models for HIV latency do not necessarily recapitulate the events occurring during viral latency *in vivo*. Compounds that efficiently increase the levels of HIV transcripts in several cell models failed to upregulate the LTR in cells isolated from virally suppressed patients [62]. To demonstrate that ING-B is able to activate latent virus *ex vivo*, CD4+ T cells were isolated from the blood of five HIV+ HAART-treated patients and incubated with ING-B, PMA or prostratin. All patients had undetectable viral load (<50 copies/mL) and CD4 counts above 500 cells/mm^3^ during blood collection (Table 1). After 24 h, cells were collected and full genome transcripts were quantitated by RT-qPCR using a set of primers and probe specific for HIV *pol*. ING-B upregulated HIV transcription in all five patients to levels similar to PMA treatment. ING-B-driven expression varied among patients with a range from 8 to 20 fold compared to vehicle control (Figure 10), indicating that ISDs are able to activate the HIV LTR in both *in vitro* and *ex vivo* primary cells.

## Discussion

Here we introduce three novel semi-synthetic PKC activators derived from ingenol diterpenes isolated from *Euphorbia tirucalli*. These compounds, named ING-A, ING-B, and ING-C, were initially evaluated in the context of HIV-LTR activation using diverse latency cell models, including five distinct J-Lat clones, ACH-2, J1.1 and U1 cells. In J-Lat cells, all ingenol synthetic derivatives (ISD) were able to efficiently induce the expression of LTR-driven GFP at concentrations as low as 0.01 µM. Despite the clonal properties of J-Lat cells, neither one of the three ISDs induced reactivation in the entire clonal population, corroborating the epigenetic regulation that is suggested to occur among these cell lines. Among the three derivatives, ING-B showed levels of HIV reactivation similar to PMA and TNF-α, and also lower cytotoxicity when compared to other diterpenes tested. Overall, all ISDs were more active than prostratin, a phorbol ester that has been previously evaluated as a potential candidate for the HIV “shock and kill” strategy. Indeed, it is required for prostratin concentrations higher than 2 µM to reactivate HIV-1 latency. Besides, high doses or long-term *in vivo* treatment with prostratin can lead to harmful side effects [35]. ISDs also led to the increase of virus production in all latency cell models here studied indicating that these compounds upregulate the expression of both early and late HIV genes, and have the potential to work in cells of both lymphocytic and monocytic origins.

Other previously isolated ingenol molecules have been used to reactivate HIV in different cell models [40]. These studies suggest that the efficiency in upregulating the HIV LTR varies depending on the nature and position of the esters in the diterpenes ring, being changes in carbon 3 and 13 the most important for PKC activation [23], [42]. The ISDs reported here, however, appear to be less cytotoxic in latently infected cell line after prolonged exposure up to 72 h.

ISDs also induced HIV-1 LTR expression in PBMCs, indicating that these compounds modulate viral production in primary cells. Upregulation of LTR-driven gene expression by ISDs in primary cells is comparable to PMA, and greater than prostratin, while exhibiting lower toxicity. A previous report shows PMA and prostratin similarly enhancing LTR-luc-expression 5 to 6 fold in PBMCs when compared to control, and in lower levels than our results. However, the concentrations of PMA and prostratin used were significantly different than in our experiments (0,04 µM for PMA and up to 10 µM for prostratin), which may explain the conflicting results [25].

It has been previously described that other ingenol diterpenes were able to block infection in cell lines, although less effectively than ZDV [63]. Ingenol 3,5,20-triacetate (ITA), for instance, was shown to inhibit HIV replication in MT-4 cells at concentrations as low as 0.05 µM [41]. The novel ISD here evaluated, while maintaining low toxicity, exhibited even lower effective concentrations than ITA when used in MT-4 cells, and were comparable to many antiretroviral compounds currently in use, including entry inhibitors, such as enfuvirtide and maraviroc [64], [65]. In addition, ING-B EC_50_ and TI values are close to classical antiretroviral compounds including ritonavir, nevirapine, delavirdine, nelfinavir and ddC [66]–[68].

The decreased surface expression of CD4, CXCR4, and CCR5 observed with ISD treatment may be an important factor in the blockade of *de novo* infection. Prostratin, PMA and bryostatin-1 downregulate CD4 and CXCR4 and present mild anti-HIV activity [25], [31], [69], and ingenol-3-angelate has been shown to downregulate surface receptors blocking *de novo* infection [70]. PBMCs treated with prostratin and infected with HIV NL4-3-Luc pseudotyped with VSV envelope were not refractory to infection despite the downregulation of CD4 and CXCR4, confirming that the control of viral replication occurred at entry level [25]. We here demonstrate that these novel ISDs are more efficient than prostratin, PMA, and bryostatin-1 in decreasing the expression of the CD4, CXCR4 and CCR5 in PBMC, leading to an almost complete blockade of both R5 and X4 HIV strains. The ability to reduce the replication of several HIV subtypes has been demonstrated using gnidimacrin [30], but not in X4 HIV strains in a dose dependent manner as we here described.

All the diterpenes evaluated in this study act mainly through PKC pathways. PKCs belong to a large and diverse family of protein kinases whose members are differentially regulated by intracellular signals such as increase in calcium concentration, diglycerides and phosphatidic acid [43]. Tigliate diterpenes such as phorbol esters activate PKC through the diglyceride-binding domain and therefore can both increase HIV-1 gene expression and also be blocked by specific PKC activators [27], [42], [71], [72]. Here we showed that the pan-PKC inhibitor GÖ6983 blocked ISD-driven GFP expression in a dose dependent manner. In similar concentrations, RO-31-8220 reduced in 75% of the ING-B-driven GFP expression, suggesting that part of the activation was modulated via PKCs α, βI, βII, γ or ε. GÖ6976, however, blocks PKC-α and PKCβI isoforms and had little effect on GFP expression, arguing that these conventional PKCs are not the main isoforms involved in ING-B-driven modulation. These results corroborate previous findings in experiments performed with prostratin and PMA in J-Lat clones 6.3 and 9.2 [35], although others, using different Jurkat derived latency models, have reported that the effect of different phorbol ester, including prostratin, PMA and P-13S, were inhibited by GÖ6976. It is possible that these differences are related to the parental Jurkat cell line used for the development of each model [73].

PKC induces latent HIV-1 gene expression by activating both NF-κB, SP-1 and AP-1 pathways [25], [74]. Prostratin effectively activates HIV gene expression in latently infected cells by stimulating IKK-dependent phosphorylation and degradation of I*κκ*B, leading to the rapid nuclear translocation of NF-κB and activation of the HIV-1 LTR [75]. Ingenol molecules have been reported to reactivate latent HIV through NF-κB and AP-1 signaling pathways, and a free hydroxyl at carbon 5 is required for PKC activation [42]. Nonetheless, our findings support that ISDs act as phorbol esters through PKC-dependent induction of NF-κB/RelA to target HIV-1 LTR expression [42], [75]. In addition, ING-B-induced downregulation of CD4, CCR5 and CXCR4 in both MT-4 and CD4+ Lymphocytes can be inhibited by GÖ6983 and RO-31-8220, indicating that PKCs also play a crucial role in downmodulation of surface markers that could interfere with viral replication [28]. The discrepancies found among these novel ISDs and other ingenol diterpenes may be related to stability, degradation, and their interaction with different isotypes of conventional or novel PKCs. Future experiments should be done to confirm whether ISDs directly interact with PKCs. We postulate, however, that ISDs might do so because of their structural and functional similarities to other bona fide PKC agonists such as PMA and DAG.

Similarly to other phorbol-like compounds, ISDs upregulate the surface expression of activation markers in T lymphocytes. Interestingly, ISDs upregulated CD69 but not CD25, although both require similar stimulation via T-cell receptors [76], [77]. CD25 is required for IL-2-driven proliferation, which suggests that cells treated with ISDs might be less sensitive to the effects of IL-2 than those treated with other diterpenes. In principle, an ideal compound for the “shock and kill” strategy should modulate the HIV-LTR without causing a broad T cell activation [8]. However, viral transcription is directly affected by events related to cell activation including translocation of the transcription NF-κB and NF-AT [4], and latency-reversing agents that do not promote cell activation appear to be ineffective in removing the virus from latency [63]. In addition, phorbol-esters also activate CD8+ T cells, which are necessary for the elimination of infected cells coming out of latency [78]. It is possible that some cell activation is even necessary for the efficacy of such compounds, provided it does not lead to systemic and harmful cytokine storms.

Most importantly, ING-B upregulated HIV transcription in CD4+ T cells isolated from virally suppressed patients. Some of these mRNAs may be originated from transcriptionally active HIV genomes found in non-resting CD4+ T cells. Therefore, future assessments should be performed to confirm that ING-B is able to activate latent HIV genomes specifically in selected resting CD4+ T cells [62]. Nonetheless, our results suggest that ISDs have the potential to reactivate latent reservoirs *in vivo*.

Our results demonstrate the dichotomous effects of novel ISDs on HIV replication: they efficiently upregulate LTR-driven transcription while downregulate surface proteins that are essential for HIV replication. Ingenol compounds have been described as strong PKC activators that uniformly upregulate HIV-LTR expression in different cell lines [79]. However, their use is limited by their high cytotoxicity and tumor-promoting properties. Here we show that novel ISDs, especially ING-B, are able to modulate HIV expression while being less cytotoxic than other PKC activators, making these molecules potential coadjuvants for the “shock and kill” therapies suggested for HIV eradication.

## Supporting Information

Figure S1 **ING-B does not induce cell proliferation, changes in cell cycle and early apoptosis.** A) Cytometry histograms showing lack of cell proliferation in CFSE-stained human PBMCs treated with 1, 5 or 10 µM ING-B for 8 days. DMSO 1% was used as negative control and IL-2/PHA as positive control. B) Graphic depicting the number of proliferating cells for each condition in three independent experiments. C) Cytometry histograms showing cell cycles in PI-stained MT-4 cells treated with ING-B or DMSO 1% (negative control). D) Graphics show the mean and standard deviation of three independent cell cycle experiments. E) Annexin V expression in PBMC isolated from two health donors and treated with ING-B 1 µM for 48 h analyzed by flow cytometry. DMSO 1% was used as vehicle control.
(TIFF)
