# Supplementary figures and images for: Dual Role of Novel Ingenol Derivatives from Euphorbia tirucalli in HIV Replication: Inhibition of De Novo Infection and Activation of Viral LTR
Source: PLoS One. 2014 May 14;9(5):e97257. doi: 10.1371/journal.pone.0097257 (PMC4020785; doi:10.1371/journal.pone.0097257)

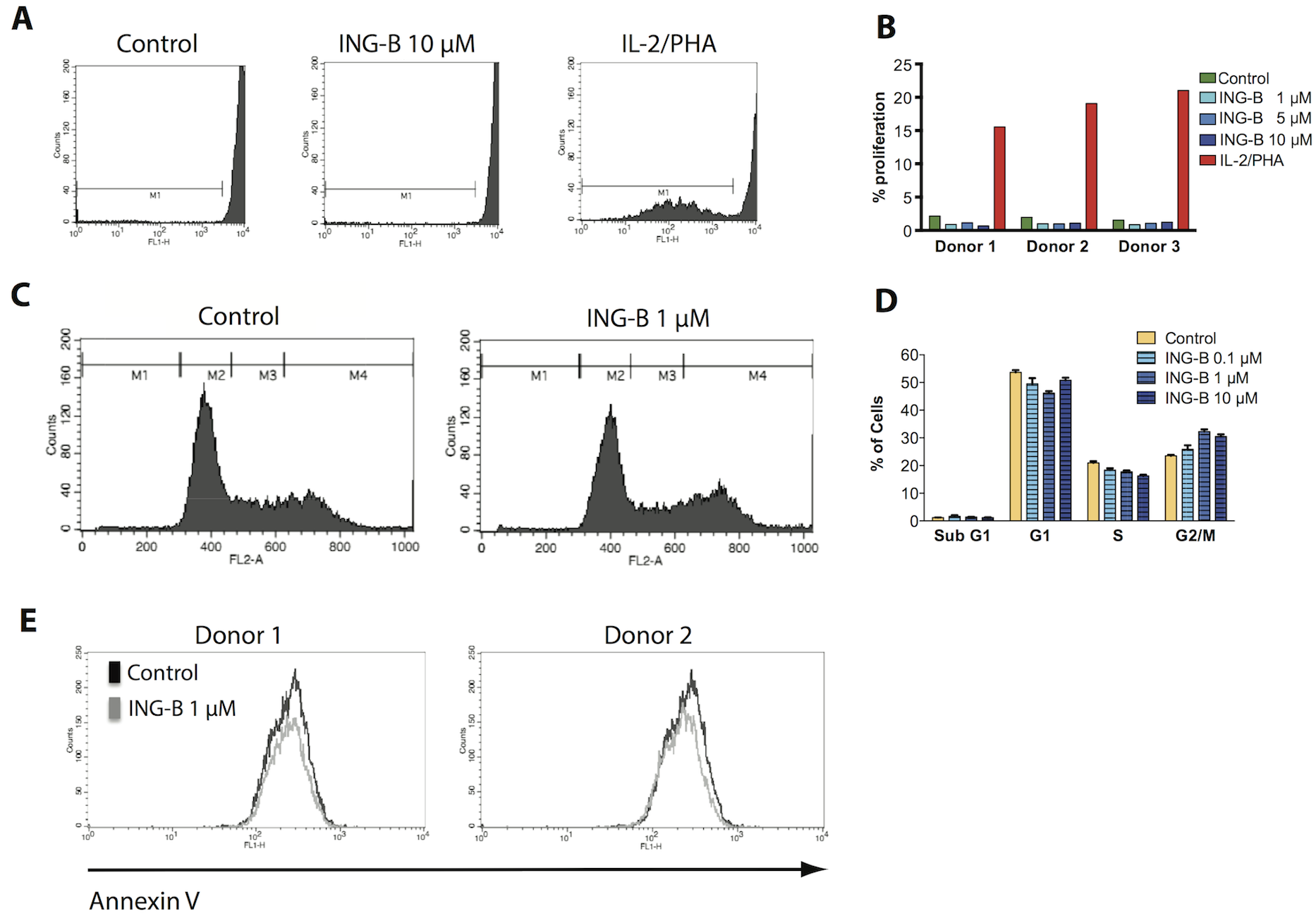

Supplement: Figure S1 — ING-B does not induce cell proliferation, changes in cell cycle and early apoptosis. A) Cytometry histograms showing lack of cell proliferation in CFSE-stained human PBMCs treated with 1, 5 or 10 µM ING-B for 8 days. DMSO 1% was used as negative control and IL-2/PHA as positive control. B) Graphic depicting the number of proliferating cells for each condition in three independent experiments. C) Cytometry histograms showing cell cycles in PI-stained MT-4 cells treated with ING-B or DMSO 1% (negative control). D) Graphics show the mean and standard deviation of three independent cell cycle experiments. E) Annexin V expression in PBMC isolated from two health donors and treated with ING-B 1 µM for 48 h analyzed by flow cytometry. DMSO 1% was used as vehicle control. (TIFF) [file pone.0097257.s001.tiff]
